# Supplementary material for: Evolution of lysine-specific demethylase 1 and REST corepressor gene families and their molecular interaction
Source: Commun Biol. 2023 Dec 14;6:1267. doi: 10.1038/s42003-023-05652-x (PMC10721905; doi:10.1038/s42003-023-05652-x)
Supplement: Supplementary file 2 — Supplementary figures [file 42003_2023_5652_MOESM2_ESM.pdf]

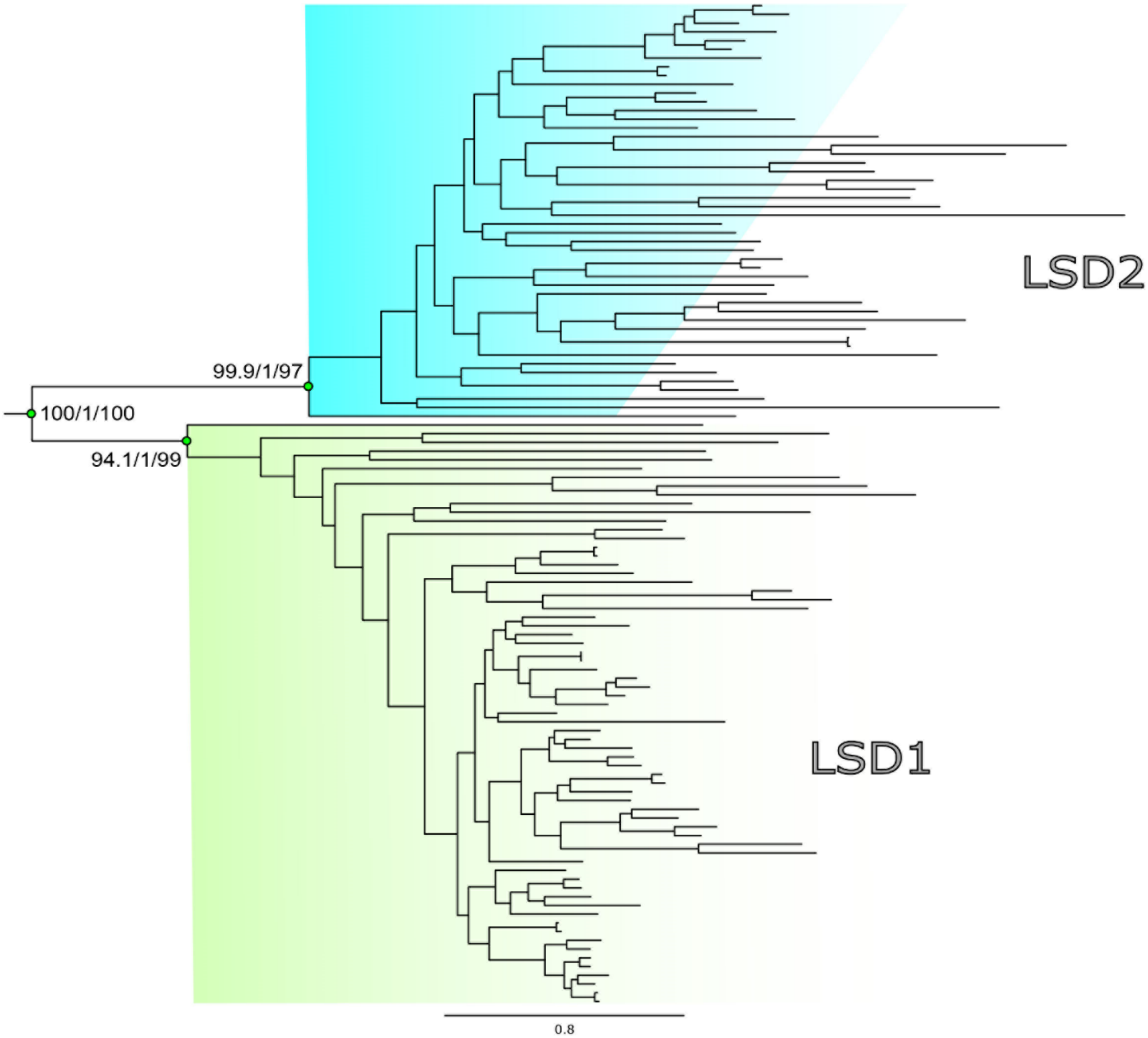

Supplementary figure 1. Maximum likelihood tree phylogram showing relationships among LSD genes of metazoa. Numbers on the nodes correspond to support from the Shimodaira-Hasegawa approximate likelihood-ratio test, approximate Bayes test, and ultrafast bootstrap values. The scale denotes substitutions per site and colors represent gene lineages. Monoamine-oxidases (MAO-A and MAO-B) sequences from human (Homo sapiens), chicken (Gallus gallus), spotted gar (Lepisosteus oculatus), and coelacanth (Latimeria chalumnae) were used as outgroup (not shown). For more details on the species included in the analysis, refer to supplementary table 4.

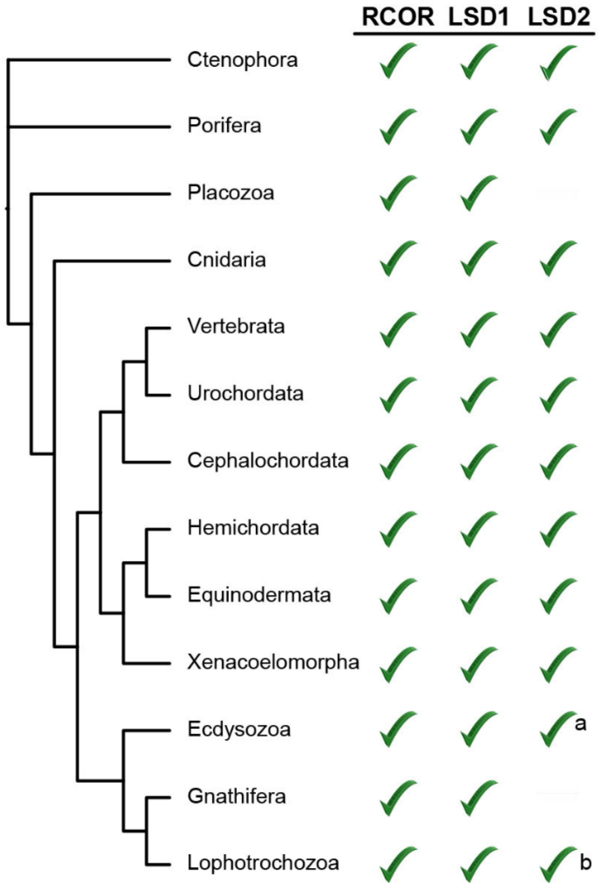

Supplementary figure 2: Phyletic distributions of RCOR and LSD genes in main groups of animals. The blue line indicates not-found sequences; aLSD2 sequences are found only in some Priapulida, Nematoda, and Arthropoda species. bLSD2 is found only in Annelida, Mollusca, Brachiopoda, Phoronida, Bryozoa, and Nemertea (detailed information in supplementary table 4).

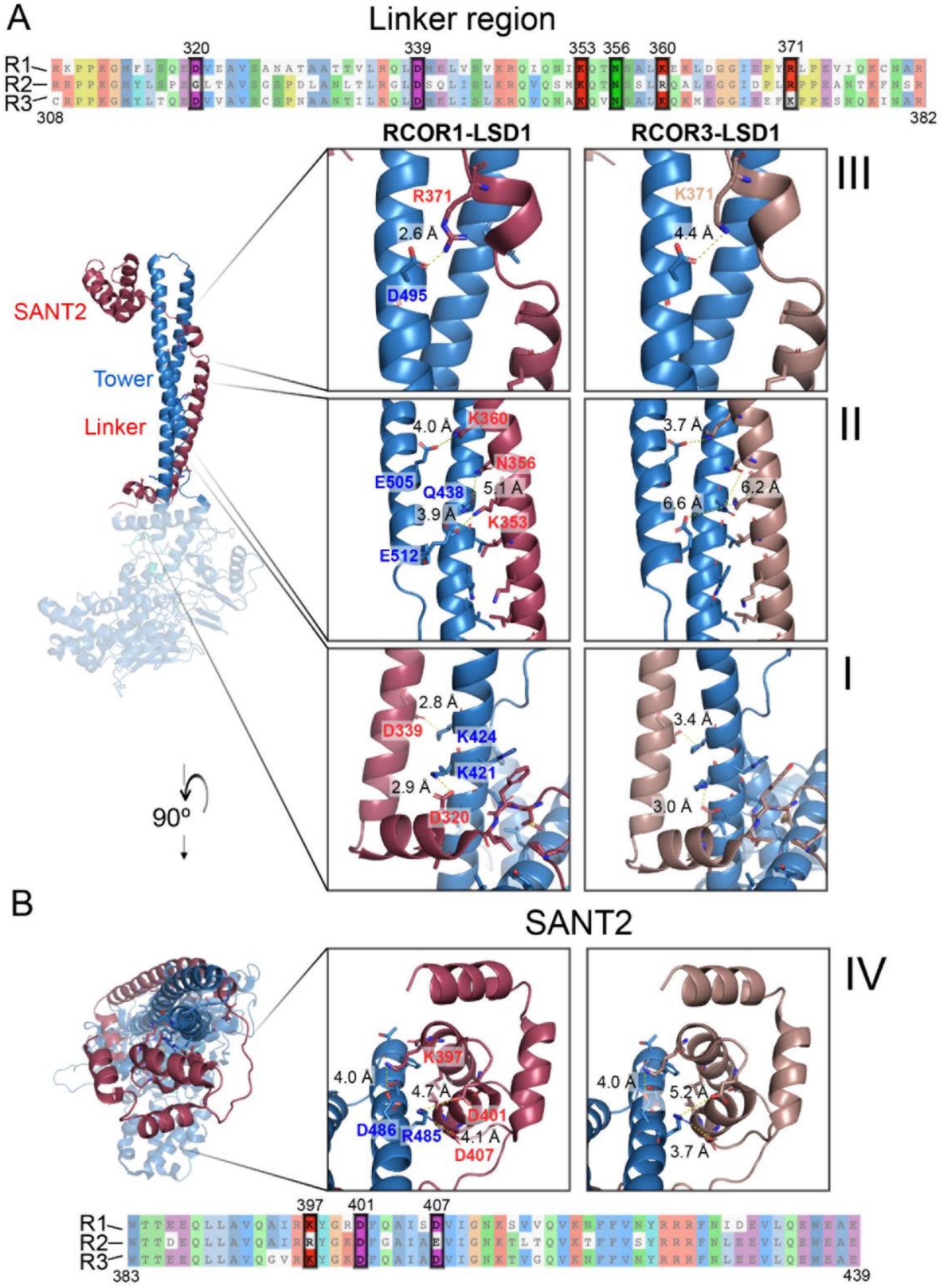

Supplementary figure 3: Comparison of the molecular details of the LSD1-RCOR1 and LSD1-RCOR3 interaction. A) (Top) Multiple sequence alignment of RCOR1, RCOR2 and RCOR3 linker domains. Highlighted positions represent RCOR interacting residues with LSD1 according to Hwang et al. 2011. (Bottom) Structure and residue interactions of LSD1-RCOR1 and LSD1-RCOR3 at different interaction interfaces (numbered I-III) of tower-linker binding. B) (Top) Structure and residue interactions of LSD1-RCOR1 and LSD1-RCOR3 at interface IV. (Bottom) Multiple sequence alignment of RCOR1, RCOR2 and RCOR3 SANT2 domain. Highlighted positions represent RCOR interacting residues with LSD1.

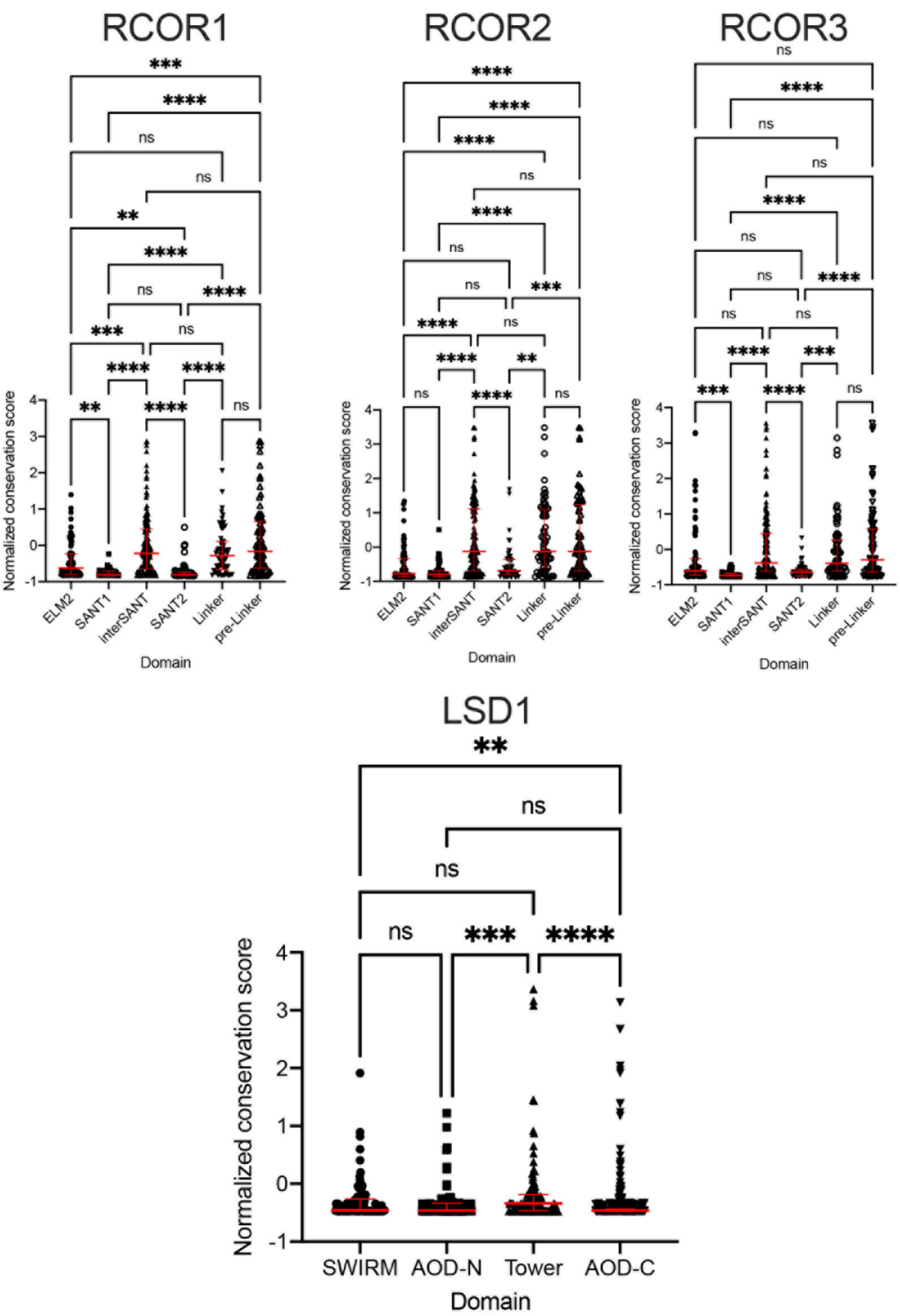

Supplementary figure 4. Statistical analyses of domain conservation of RCORs' and LSD1 in jawed vertebrates. Statistics of ConSurf continuous conservation scores of each protein domain in comparison to every other domain found in the same protein. We performed the Kruskal-Wallis nonparametric test with posterior Dunn's multiple comparisons test. We report the p-value of each comparison in the corresponding section of the manuscript. Top left: RCOR1, Top middle: RCOR2, Top right: RCOR3, Bottom: LSD1. Error bars represent the median with an interquartile range.

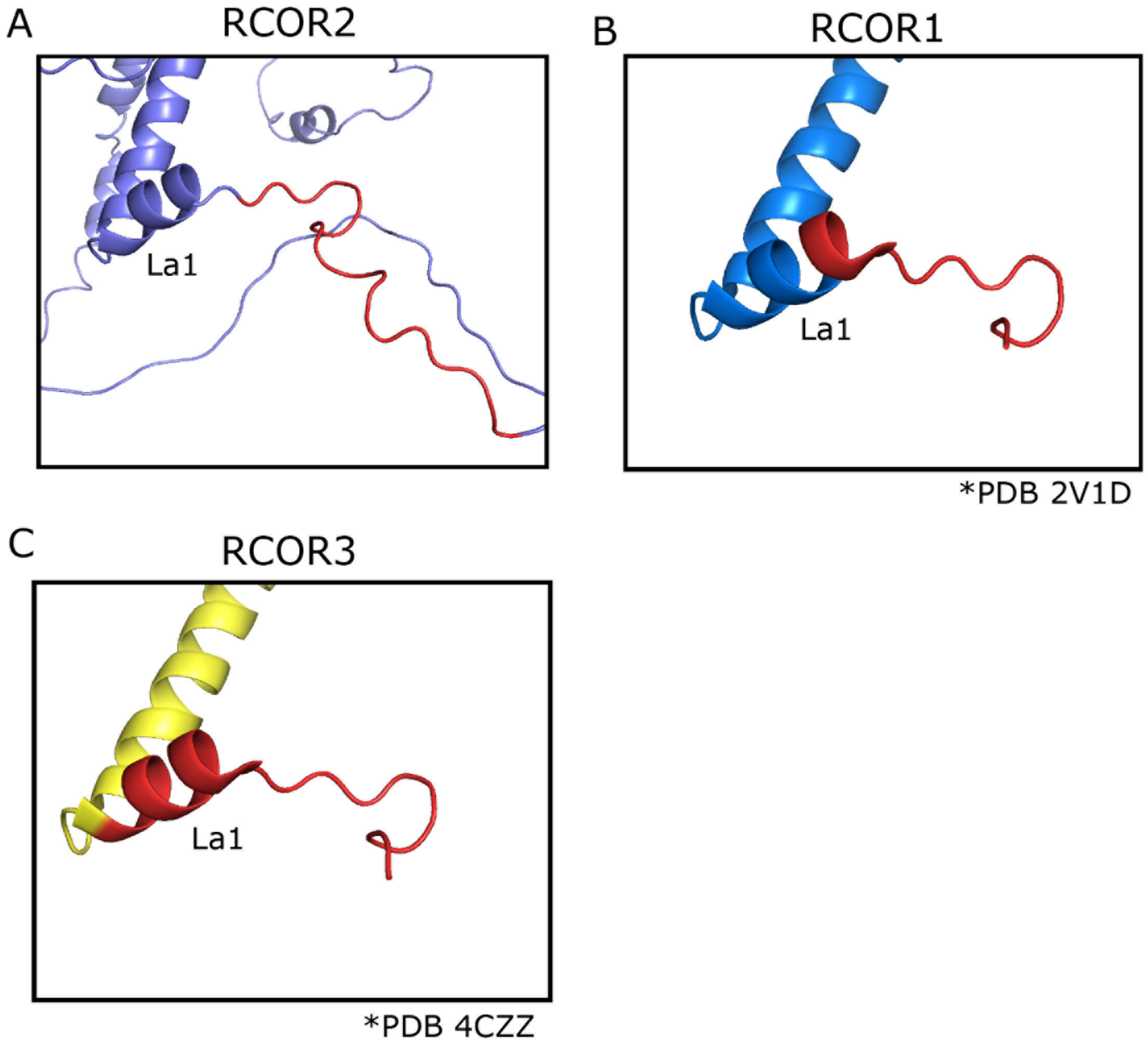

Supplementary figure 5. Structural representation of a highly conserved section in RCOR1, RCOR2 and, RCOR3 among jawed vertebrates. (A) The red color represents highly conserved residues that precede the La1 helix in RCOR2. (B) The red color depicts a highly conserved patch with amino acids preceding and including the La1 helix in RCOR1. (C) The red color represents highly conserved amino acids that precede and include the La1 helix in RCOR3. RCOR2s' structure corresponds to an AlphFold prediction of human RCOR2. RCOR1's and RCOR3s' structure correspond to PDB codes 2V1D and 4CZZ.

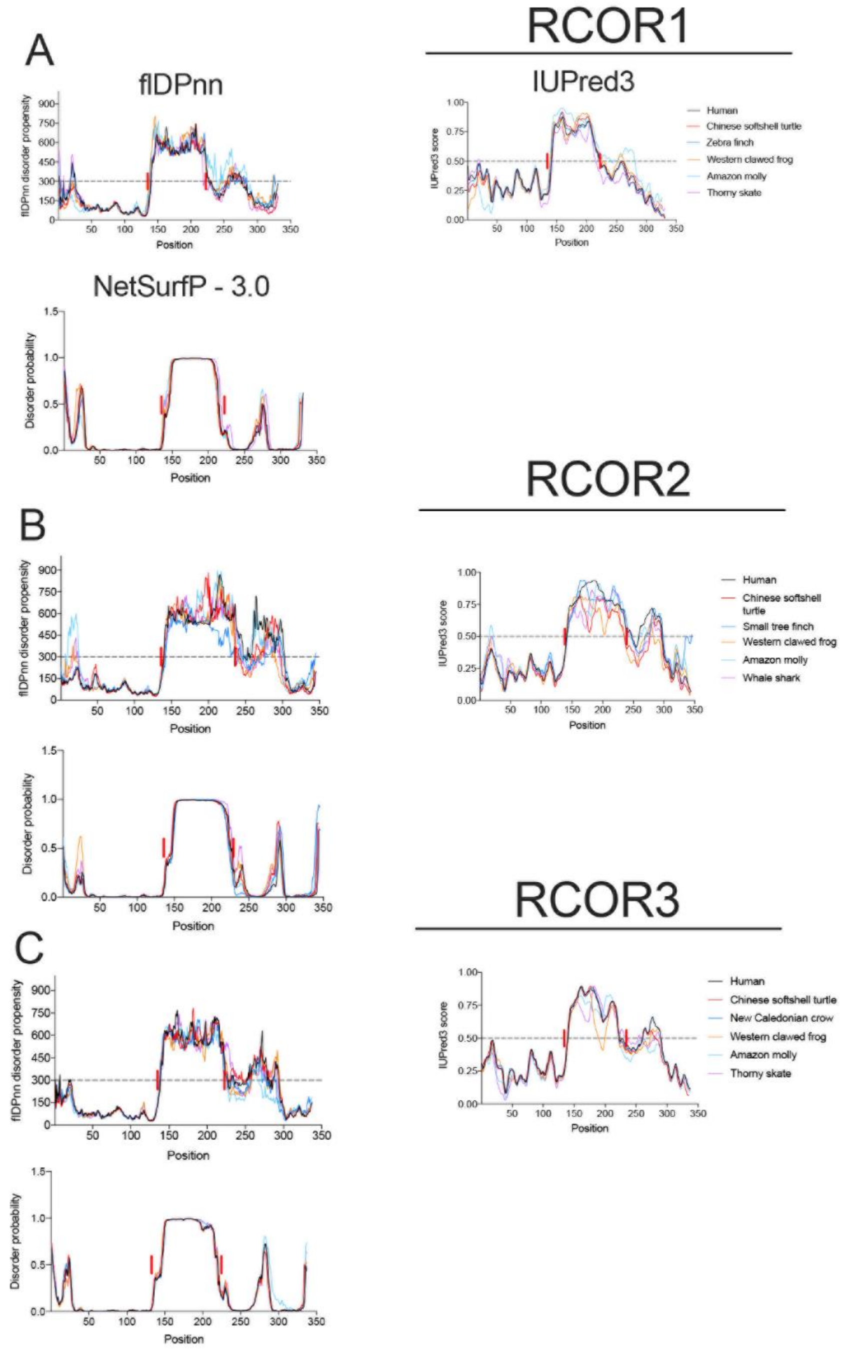

Supplementary figure 6. IDR predictions of jawed-vertebrate RCORs' pre-linker region. RCOR1, RCOR2, and RCOR3 sequences are from representative species of the main groups of vertebrates (mammals: human, reptiles: Chinese softshell turtle, birds: zebra finch, small tree finch, and new Caledonian crow, amphibians: western clawed frog, bony fish: amazon molly, cartilaginous fish: thorny skate and whale shark) were submitted to IDR prediction using flDPnn, IUPred3 and NetSurf - 3.0. Vertical red lines in each graph represent the limits of the pre-linker region.

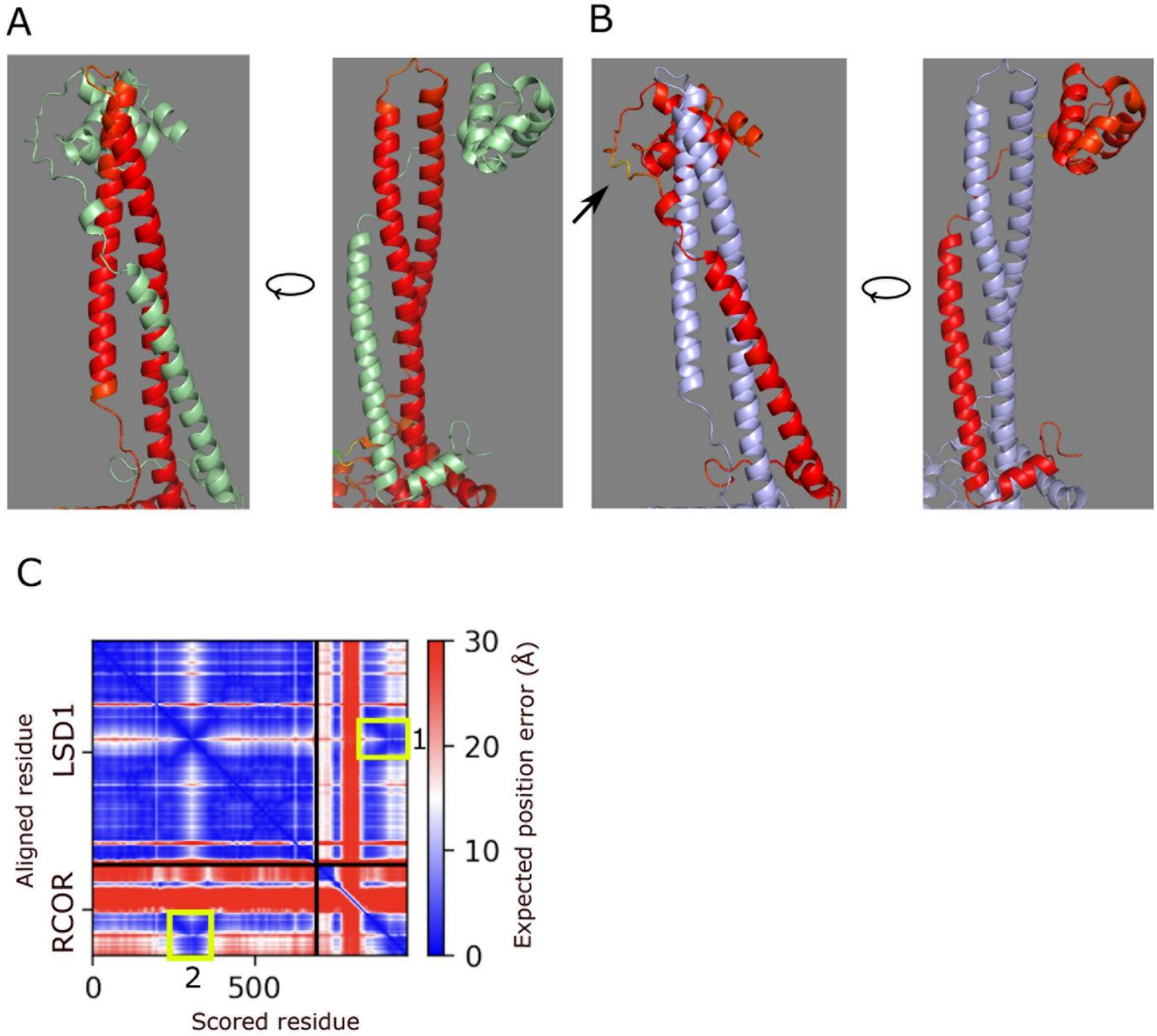

Supplementary figure 7. Local model confidence and domain-relative position in the ancestral LSD1-RCOR complex. (A-B) Predicted local-distance difference test (pLDDT) of ancestral LSD1 (A) and RCOR (B). Red: pLDDT > 90. The black arrow denotes a region of lower local model confidence. (C) Predicted aligned error (PAE) plot showing good inter-domain accuracy of RCORs' linker domain with respect to LSD1s' tower aligning (yellow box, 1) and LSD1s' tower domain with RCORs' linker domain aligning (yellow box 2).
