# Supplementary material for: Evolution of lysine-specific demethylase 1 and REST corepressor gene families and their molecular interaction
Source: Commun Biol. 2023 Dec 14;6:1267. doi: 10.1038/s42003-023-05652-x (PMC10721905; doi:10.1038/s42003-023-05652-x)
Supplement: Supplementary file 3 — Description of additional supplementary files [file 42003_2023_5652_MOESM3_ESM.docx]

Description of Additional Supplementary Files

**File name:** Supplementary Data 1**-**6

**Description:** Tables detailing sequence access codes

**File name**: Supplementary Data 7

**Description:** The source data behind the graphs in the paper
